# Ductular reaction as a spatial fibrogenic niche: cholangiocyte–macrophage–fibroblast crosstalk in chronic liver disease

**DOI:** 10.3389/fmed.2026.1987203

**Published:** 2026-10-08

**Authors:** Kun Wang, Linjun Li, Xiangkun Mei, Hongqiang Gao

**Affiliations:** 1 Department of General Hepatobiliary Surgery, Ganmei Hospital Affiliated to Kunming Medical University, Kunming, China; 2 Institute of Reproductive Health, Tongji Medical College, Huazhong University of Science and Technology, Wuhan, China

**Keywords:** cholangiocyte, ductular reaction, fibrosis, hepatic stellate cell, macrophage, portal fibroblast, single-cell transcriptomics, spatial niche

## Abstract

Ductular reaction—the expansion of reactive bile ductules at the portal–parenchymal interface—has traditionally been interpreted as a proliferative or regenerative response of the biliary epithelium to chronic injury. Here we argue that this histological phenomenon is better understood as a spatial fibrogenic niche: a physically organized, self-reinforcing microenvironment in which reactive cholangiocytes, macrophages, and matrix-producing mesenchymal cells engage in bidirectional signaling that converts repair into fibrosis. We integrate single-cell and spatial transcriptomic evidence showing that reactive cholangiocytes act as a signaling hub, secreting TGF-*β*, PDGF, CCL2, CXCL chemokines, IL-6, osteopontin, and Hedgehog ligands; that recruited and tissue-resident macrophages amplify this axis; and that portal fibroblasts and hepatic stellate cells close a feed-forward loop by depositing extracellular matrix and growth factors that sustain the niche. We propose that a cholangiocyte–macrophage–fibroblast feed-forward loop, stabilized by chemokine-driven recruitment, TGF-*β*-centered fibrogenesis, mechanical feedback, and senescence, underlies ductular-associated fibrosis across cholestatic and metabolic liver diseases. Targeting the pathological organization of this niche—rather than any single cell type—may offer a new therapeutic framework for chronic liver fibrosis.

## 1 Introduction

Ductular reaction—the expansion of reactive bile ductules at the interface between the portal tract and the hepatic parenchyma—is among the most common and reproducible histological findings in chronic liver disease. It is observed in cholestatic disorders, including primary sclerosing cholangitis (PSC), primary biliary cholangitis (PBC), and biliary atresia, but it is equally prominent in parenchymal injury of metabolic, alcoholic, and viral etiology (1, 2). Identified by cytokeratin 7 (CK7), cytokeratin 19 (CK19), SOX9, and EpCAM, the reaction expands reactive biliary cells together with a surrounding inflammatory and mesenchymal cuff, and its extent has long served as a histological surrogate of disease activity and fibrosis stage (1, 2). The near-universality of ductular reaction across etiologically distinct diseases is itself a clue that it reflects a shared, fundamental response of the liver to chronic injury rather than a disease-specific epiphenomenon.

Traditionally, ductular reaction has been interpreted primarily as a proliferative or regenerative response of the biliary epithelium—an attempt by the liver to replenish damaged bile ducts and, in severe injury, to supply hepatocytes through the activation of hepatic progenitor-like cells (3). This regenerative reading is attractive, and it is certainly part of the story: ductular cells are bipotential in many models and can contribute to parenchymal restoration. It is, however, incomplete. A substantial body of evidence now indicates that ductular reaction is not a uniform epithelial compartment but a spatially organized assembly of reactive cholangiocytes, macrophages, portal fibroblasts, hepatic stellate cells, endothelial cells, and extracellular matrix (ECM), whose coordinated signaling actively drives—rather than merely accompanies—fibrogenesis (4–6). Correlatively, the magnitude of ductular reaction tracks fibrosis stage and adverse outcomes across diseases, even where it was once dismissed as a passive bystander of scarring (7).

This review proposes a “ductular reaction-associated spatial fibrogenic niche” framework. We synthesize evidence that reactive cholangiocytes operate as the signaling hub of this niche, that macrophages amplify ductular fibrogenesis, and that fibroblasts and hepatic stellate cells provide the matrix-producing arm, and we argue that the spatiotemporal interplay among these three compartments—rather than any one cell type in isolation—constitutes the engine of chronic hepatic fibrosis. We then consider how this niche manifests in specific diseases, how emerging spatial technologies are redefining it, and how targeting its organization, rather than its individual cells, might yield new antifibrotic strategies. The overall conceptual framework of the ductular reaction-associated spatial fibrogenic niche, including the transition from adaptive repair to progressive fibrosis, is summarized in Figure 1.

**Figure 1:**
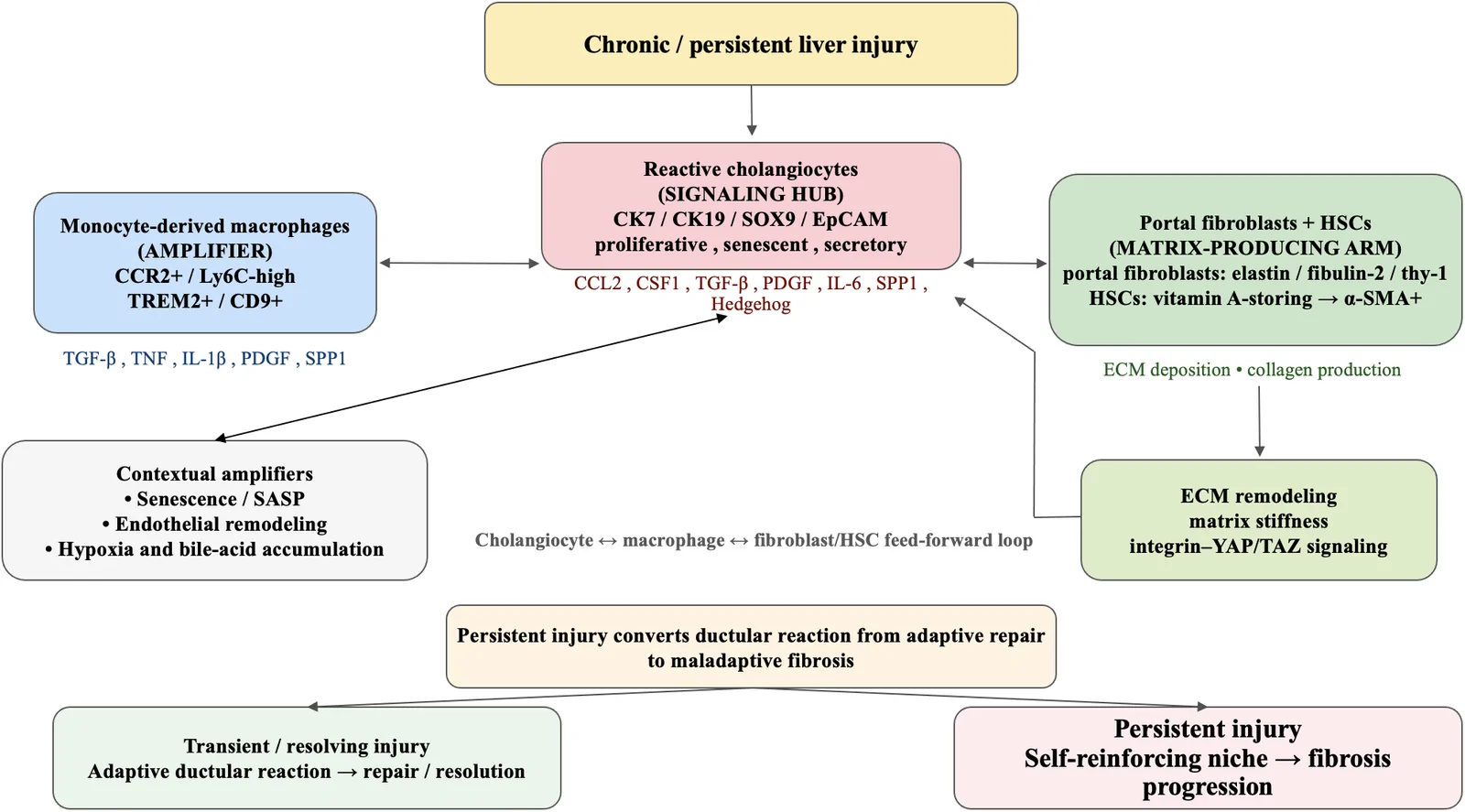
Ductular reaction as a spatial fibrogenic niche in chronic liver disease. Persistent liver injury shifts the ductular reaction from an adaptive regenerative response toward a self-reinforcing fibrogenic niche organized around reactive cholangiocytes, macrophages, portal fibroblasts, and hepatic stellate cells (HSCs). Reactive cholangiocytes function as a signaling hub by releasing CCL2, CSF1, TGF-*β*, PDGF, IL-6, osteopontin (SPP1), and Hedgehog ligands, thereby recruiting and reprogramming macrophages and activating mesenchymal cells. Macrophages amplify the niche through TGF-*β*, TNF, IL-1β, PDGF, and SPP1, whereas portal fibroblasts and HSCs deposit extracellular matrix (ECM) and reinforce epithelial and immune activation. ECM remodeling and matrix stiffening further stabilize fibrogenic signaling through integrin-linked mechanotransduction and context-dependent YAP/TAZ signaling. Senescence-associated signaling, endothelial remodeling, hypoxia, and bile-acid accumulation provide additional contextual amplification. Together, these reciprocal interactions promote the transition from adaptive repair toward persistent fibrosis.

## 2 Ductular reaction: from histological phenomenon to dynamic fibrogenic niche

Ductular reaction denotes the expansion of reactive bile ductules and their associated cellular constituents at the portal–parenchymal interface in response to liver injury (1). Histologically it encompasses a spectrum, from simple proliferation of pre-existing bile ductules to the appearance of atypical, frequently irregular and anastomosing tubular structures lined by cells that co-express biliary (CK7, CK19, SOX9, EpCAM) and progenitor markers (8). This morphological spectrum reflects underlying biological heterogeneity: some ductular profiles represent genuine regenerative attempts, whereas others are disorganized, non-functional structures that fail to reconnect the biliary tree and instead accumulate within an expanding scar (1, 2). The reaction is invariably accompanied by periductular inflammation, deposition of ECM, and expansion of a loose mesenchymal cuff of activated fibroblasts—features that already hint at its intimate coupling to fibrogenesis (2).

Ductular reaction is a near-universal feature of chronic liver disease, but its prominence and character differ by etiology. It is most florid in cholestatic conditions—PSC, PBC, and biliary atresia—where cholangiocyte injury is the primary insult (1, 2). It is also prominent, however, in advanced metabolic dysfunction-associated steatohepatitis (MASH), alcohol-associated liver disease, and chronic viral hepatitis, where it emerges late and tracks the transition to bridging fibrosis (7). This cross-etiology convergence is important for the argument developed here: if the same reactive biliary structures appear in diseases whose primary insults are so different—autoimmune, cholestatic, metabolic, toxic, and viral—then the biology they share is likely to reside not in the initial injury but in a conserved downstream program of multicellular interaction.

The cellular sources of ductular reaction have been intensely debated, and three contributors are consistently supported. First, resident cholangiocytes proliferate and acquire a reactive phenotype in response to biliary injury. Second, hepatic progenitor-like cells—the oval cells of rodents—residing within the canals of Hering expand bipotentially when hepatocyte replication is impaired. Third, mature hepatocytes can transdifferentiate toward a biliary fate through hepatocyte-to-biliary plasticity, a process dependent on Notch and Hippo/YAP signaling that is particularly prominent in biliary-dominant injury (3, 9, 10). The relative contribution of each source varies by disease context, species, and injury severity, and this cell-of-origin question remains unresolved. We deliberately do not adjudicate it here, because it does not materially change the niche argument: whatever their origin, the epithelial cells of the ductular reaction converge on a shared reactive, secretory, pro-fibrotic phenotype that organizes the surrounding microenvironment.

The central proposition of this review is that ductular reaction is not a pure epithelial phenomenon but a spatial niche. Reactive cholangiocytes are not surrounded by a single cell type; they are encircled by a stereotyped ensemble of macrophages, portal fibroblasts, hepatic stellate cells, liver sinusoidal endothelial cells, and a growing ECM scaffold (4–6). This is not a loose association but a structured neighborhood. Spatial profiling has shown that these cells are organized into periductular zones with defined gradients of signaling molecules and matrix components, and that specific cell states—scar-associated macrophages, activated myofibroblasts, and reactive cholangiocytes—are reproducibly co-localized around the same ductular profiles (5).

Why does this spatial organization matter, rather than merely the presence of these cells somewhere in the liver? Because proximity is functional. Confining ligands and their receptors to a narrow periductular band concentrates signaling, enforces cell–cell contact, and creates mechanical constraints that feed back onto gene expression through integrins and mechanosensitive pathways. A cholangiocyte that secretes CCL2, a monocyte that follows that gradient, and a fibroblast that stiffens the local matrix are each individually unremarkable; placed within tens of micrometers of one another, they constitute a self-reinforcing circuit. We therefore propose that the ductular reaction-associated niche—defined by the spatial co-organization of reactive cholangiocytes, immune cells, mesenchymal cells, and ECM—is the functional unit through which chronic injury is translated into fibrosis.

## 3 Reactive cholangiocytes as the signaling hub of the fibrogenic niche

Reactive cholangiocytes are far more than hyperplastic bile-duct cells. Under chronic injury they undergo a profound phenotypic remodeling that encompasses proliferation, a neuroendocrine and secretory phenotype, upregulation of pro-inflammatory cytokines, and—critically—entry into senescence (6, 11). This remodeling is graded. Mild, transient injury elicits an adaptive, proliferative response that restores epithelial integrity and then resolves. Persistent injury, by contrast, drives an inflammatory, senescent, and secretory state that is intrinsically pro-fibrotic and that no longer serves a reparative function (1). The reactive cholangiocyte is therefore best characterized not by a single marker but by a constellation of proliferation, senescence, inflammation, and secretion—and it is this constellation, rather than proliferation alone, that defines its role in the niche.

The functional consequence of this remodeling is that reactive cholangiocytes become a dominant source of fibrogenic and chemotactic signals, acting on every other cellular compartment within the periductular space. Several classical pathways stand out. Reactive cholangiocytes secrete TGF-*β*—notably the TGF-*β*2 isoform—a master driver of mesenchymal activation that acts directly on portal fibroblasts and hepatic stellate cells (1, 6). They secrete PDGF, in particular PDGF-BB, which recruits and activates those same mesenchymal cells and promotes their proliferation and migration (1). They secrete CCL2 (MCP-1), the principal monocyte chemoattractant of the injured liver, together with a panel of CXCL chemokines that broaden the inflammatory infiltrate (12). They secrete IL-6, which in autocrine fashion sustains cholangiocyte proliferation and in paracrine fashion promotes inflammation (1). They secrete osteopontin (SPP1), which polarizes macrophages toward a pro-fibrotic phenotype (13). And they secrete Hedgehog ligands—Shh and Ihh—which activate a dedicated population of peribiliary mesenchymal cells (14, 15). Together, this secretory program supports a central organizing role for reactive cholangiocytes in initiating and sustaining the niche's inflammatory and fibrogenic signaling.

The Hedgehog axis deserves emphasis because it is one of the clearest demonstrations that cholangiocyte-derived signals are spatially targeted rather than diffuse. In biliary injury, Hedgehog-responsive peribiliary mesenchymal cells accumulate precisely around reactive ductules, and genetic or pharmacologic inhibition of the pathway attenuates biliary fibrosis (14, 15). This is, in essence, a niche-specific ligand–receptor system: the cholangiocyte produces the ligand, and a dedicated mesenchymal responder that resides immediately adjacent to it transduces the signal into matrix production.

These observations connect repair directly to fibrosis through a transition that we regard as pivotal: adaptive regeneration → persistent injury → maladaptive ductular reaction → fibrosis. In its adaptive phase, ductular reaction restores biliary continuity and is self-limiting. When injury persists, the same program fails to resolve: cholangiocytes remain locked in a proliferative-secretory state, continue to emit fibrogenic and chemotactic signals, and recruit a self-perpetuating inflammatory–mesenchymal response (1, 6). Cholangiocyte-intrinsic signaling such as the non-canonical NF-*κ*B mediator NIK directly sustains ductular reaction and fibrosis, indicating that the epithelial compartment itself drives, rather than merely reports, the fibrogenic program (16). The ductular reaction thereby converts from a repair structure into a fibrogenic niche—a conceptual shift, from “reaction” as outcome to “niche” as driver, that is central to our framework.

## 4 Macrophages as amplifiers of ductular fibrogenesis

Hepatic macrophages comprise two broad populations whose origins, maintenance, and functions differ. Resident Kupffer cells arise from embryonic precursors and self-renew locally, whereas monocyte-derived macrophages are recruited from circulating Ly6C-high monocytes through the CCL2–CCR2 and CSF1 axes during injury (17, 18). This distinction is not merely academic: it maps onto divergent roles. In chronic injury the monocyte-derived compartment expands substantially and becomes the principal source of pro-fibrotic mediators, while Kupffer cells can also contribute—particularly in cholestatic contexts, where they are positioned at the biliary interface (18, 19). The balance between these populations, and their phenotypic conversion upon entering the niche, is a major determinant of whether injury resolves or progresses.

Communication between cholangiocytes and macrophages is bidirectional and central to niche amplification. Reactive cholangiocytes recruit macrophages by secreting CCL2 and CSF1, thereby initiating the immune arm of the niche (12, 20). In return, macrophages release TGF-*β*, TNF, IL-1β, PDGF, and osteopontin, which further stimulate ductular reaction and fibroblast activation (13, 19). This creates a self-amplifying loop in which the cholangiocyte summons the very cells that will drive its own reactive phenotype and the surrounding fibrogenesis. The macrophage is therefore not a bystander but an amplifier of ductular fibrogenesis.

A particularly compelling illustration comes from osteopontin. Bile-duct-associated macrophages that express osteopontin are enriched in fibrotic cholestatic livers and correlate with fibrosis, suggesting that a distinct macrophage population is anatomically and functionally tethered to the biliary niche (13). Because osteopontin itself promotes macrophage recruitment and polarization and has been implicated across a broad range of chronic liver diseases, it exemplifies a niche-reinforcing factor that operates at multiple nodes of the loop (13, 21).

Single-cell studies have resolved macrophages into dynamically changing states that map onto disease progression. In fibrotic human cirrhosis, scar-associated macrophages (SAMacs)—marked by TREM2 and CD9—accumulate within the fibrotic niche and express pro-fibrotic factors (4). Related TREM2-positive lipid-associated macrophages (22) and CD9-positive macrophages define conserved niches across human liver disease (5). An inflammatory, monocyte-derived macrophage population predominates in early steatohepatitis, whereas a more restorative or scar-associated phenotype accumulates as fibrosis advances (18, 21). These state transitions—from inflammatory to scar-associated—provide a molecular timeline linking macrophage phenotype to niche evolution, and they suggest that the niche does not merely accumulate macrophages but actively reprograms them.

## 5 Fibroblasts and hepatic stellate cells as the matrix-producing arm

Portal fibroblasts are the principal mesenchymal population associated with the ductular reaction in cholestatic disease. They reside in the portal mesenchyme and are activated early in biliary injury, where they differentiate into collagen-producing myofibroblasts (23). They are distinguished from hepatic stellate cells by markers including elastin, fibulin-2, and thy-1, and—importantly—a subset with mesenchymal stem cell features appears to constitute a reservoir of proliferative myofibroblasts in biliary fibrosis (23). This positions portal fibroblasts as the matrix-producing arm most tightly coupled to the periductular niche, and it explains why biliary fibrosis classically begins as periductular, concentric scarring rather than as the sinusoidal fibrosis typical of parenchymal disease.

Hepatic stellate cells (HSCs), by contrast, are the dominant myofibroblast source in chronic parenchymal injury of metabolic, alcoholic, and viral etiology (24, 25). Upon activation, quiescent vitamin A-storing HSCs transdifferentiate into *α*-SMA-positive myofibroblasts that deposit fibrillar ECM and secrete growth factors (26). Although classically associated with sinusoidal injury, HSCs also contribute to portal and bridging fibrosis, and their activation is intimately linked to the inflammatory milieu of the ductular niche (24). The two mesenchymal lineages are therefore not mutually exclusive but complementary: portal fibroblasts dominate the biliary and periductular compartment, and HSCs dominate the sinusoidal and septal compartment, with both converging on the same activated myofibroblast effector program in advanced disease.

Single-cell transcriptomics has dissolved the notion of a homogeneous “fibroblast.” Analyses of human and murine liver reveal distinct portal fibroblast subsets, a continuum of activated myofibroblasts, and specialized scar-associated mesenchymal populations, with functionally opposing HSC subpopulations distinguished in hepatocarcinogenesis (27–29). This heterogeneity carries a functional implication for the niche concept: different mesenchymal subsets serve different niches, and therapeutic strategies that target “fibroblasts” as a single entity are likely to miss the subset that actually sustains a given periductular lesion.

The cholangiocyte–fibroblast axis closes a genuine positive-feedback loop. Reactive cholangiocytes activate fibroblasts through TGF-*β*, PDGF, and Hedgehog ligands, inducing collagen production (1, 15). In return, fibroblasts deposit ECM and secrete growth factors that sustain cholangiocyte proliferation and the reactive phenotype, physically embedding the ductular cells in a stiffening, pro-inflammatory matrix (1, 30). This bidirectionality is the mechanistic core of the niche concept: neither compartment persists in its pathological state without the other.

Together, these three sections establish the cast of the niche: a cholangiocyte hub that emits fibrogenic and chemotactic signals, a macrophage amplifier that reprograms in response to those signals, and a mesenchymal effector arm that converts the signals into matrix and, in turn, sustains the epithelial and immune compartments. Table 1 summarizes the defining markers, cell states, and functional contributions of the cellular compartments of the niche. What remains is to explain how these three actors are wired into a single self-reinforcing circuit—the subject of the next section.

**Table 1:** Key cellular compartments of the ductular fibrogenic niche.

Compartment	Key markers/cell states	Function in the niche
Reactive cholangiocytes	CK7, CK19, SOX9, EpCAM; proliferative, senescent, secretory	Signaling hub; secretes TGF-*β*, PDGF, CCL2, IL-6, SPP1, Hedgehog ligands
Monocyte-derived macrophages	CCR2+ Ly6C-high; TREM2+, CD9 + scar-associated	Amplify fibrogenesis; release TGF-β, TNF, IL-1β, PDGF, SPP1
Resident Kupffer cells	Embryo-derived, self-renewing	Contribute at the biliary interface in cholestasis
Portal fibroblasts	Elastin, fibulin-2, thy-1; MSC-like subset	Periductular myofibroblasts; collagen production
Hepatic stellate cells	Vitamin A-storing; *α*-SMA after activation	Sinusoidal/septal myofibroblasts in parenchymal injury
Liver sinusoidal endothelial cells	Capillarized in injury	Gate inflammation; laminin deposition
Extracellular matrix	Collagen, laminin	Mechanical feedback via integrin-linked mechanotransduction; context-dependent YAP/TAZ signaling

CK7, cytokeratin 7; CK19, cytokeratin 19; SOX9, SRY-box transcription factor 9; EpCAM, epithelial cell adhesion molecule; TGF-β, transforming growth factor-beta; PDGF, platelet-derived growth factor; CCL2, C-C motif chemokine ligand 2; IL-6, interleukin-6; SPP1, secreted phosphoprotein 1; CCR2, C-C chemokine receptor type 2; TREM2, triggering receptor expressed on myeloid cells 2; CD9, cluster of differentiation 9; MSC, mesenchymal stem cell; α-SMA, alpha-smooth muscle actin; ECM, extracellular matrix; YAP/TAZ, yes-associated protein/transcriptional coactivator with PDZ-binding motif.

## 6 Building the spatial fibrogenic niche: multicellular crosstalk and feed-forward loops

The preceding sections describe the three actors separately; here we reassemble them. We propose that the niche is sustained by a triangular, self-reinforcing loop—reactive cholangiocyte ↔ macrophage ↔ fibroblast ↔ reactive cholangiocyte—that we term the cholangiocyte–macrophage–fibroblast feed-forward loop. The loop has several separable but interlocking components, which we dissect in turn.

Chemokine-mediated inflammatory recruitment initiates and maintains the loop. Reactive cholangiocytes secrete CCL2 and CXCL chemokines that recruit monocytes and other inflammatory cells into the periductular space (1, 12). Recruited monocytes differentiate into pro-fibrotic macrophages that in turn secrete additional chemokines, sustaining recruitment and reinforcing the niche's inflammatory tone (17, 18). Recruitment is thus not a one-time event but a self-perpetuating process that keeps the niche continuously populated with effector cells.

TGF-*β*-centered fibrogenic signaling sits at the center of the loop. Produced by both reactive cholangiocytes and recruited macrophages, TGF-*β* activates portal fibroblasts and HSCs to produce ECM and further TGF-*β*, creating a fibrogenic auto-induction circuit (6, 25, 26). TGF-*β* also directly sustains cholangiocyte proliferation and epithelial–mesenchymal-transition-like changes in biliary injury (14). The convergence of epithelial- and macrophage-derived TGF-*β* on the same mesenchymal targets—together with the latent TGF-*β* reservoir embedded in the newly deposited matrix—is a hallmark of the niche and a reason it is difficult to interrupt with single-agent blockade.

ECM remodeling and mechanical feedback make the matrix an active participant rather than an inert output. Fibroblast-deposited collagen and laminin stiffen the periductular stroma, and this altered mechanical environment may engage integrin-dependent mechanotransduction pathways, including context-dependent YAP/TAZ signaling, in cholangiocytes and mesenchymal cells (31). Integrin *α*v*β*6 on injured epithelium can activate latent TGF-*β*1, providing a mechanistic link between matrix adhesion and fibrogenic signaling (32); however, the magnitude and cell-type specificity of these pathways in the ductular niche remain to be defined. The niche may therefore acquire a form of mechanical memory in which matrix stiffening helps sustain the signaling environment that produced it.

Senescence and SASP-mediated niche stabilization convert a potentially reversible response into a persistent one. Senescent cholangiocytes—prominent in cholestatic disease—secrete a senescence-associated secretory phenotype (SASP) of pro-inflammatory and pro-fibrotic factors that sustains inflammation and fibrosis (11, 33). Senescent and p21-positive TREM2-positive macrophage populations similarly reinforce the niche, and the SASP provides a mechanistic link between chronic injury and the persistence of fibrogenesis even after the initial insult recedes (34).

Vascular and metabolic contributions support, but do not dominate, the niche. Liver sinusoidal endothelial cells undergo capillarization and gate the inflammatory response, and laminin deposition by endothelial cells shapes cholangiocyte fate (35, 36). Local hypoxia and bile-acid accumulation further condition the niche, promoting cholangiocyte proliferation and inflammation (6). We treat these inputs as contextual amplifiers rather than core drivers: they tune the set-point of the loop, but the loop itself is defined by the epithelial–immune–mesenchymal interactions described above. Table 2 summarizes the principal intercellular signaling axes of the niche, specifying the source and target cell for each and its effect within the feed-forward loop.

**Table 2:** Core intercellular signaling axes within the ductular fibrogenic niche.

Signaling axis	Source cell	Target cell	Effect in the niche
CCL2–CCR2	Reactive cholangiocytes	Monocyte-derived macrophages	Inflammatory recruitment
CSF1–CSF1R	Reactive cholangiocytes	Macrophages	Macrophage survival and polarization
TGF-β (β1/β2)	Cholangiocytes, macrophages	Portal fibroblasts, HSCs	ECM production and activation
PDGF-BB	Reactive cholangiocytes	Portal fibroblasts, HSCs	Proliferation and migration
Hedgehog (Shh/Ihh)	Reactive cholangiocytes	Peribiliary mesenchymal cells	Fibrogenic activation
Osteopontin (SPP1)	Macrophages, cholangiocytes	Macrophages, fibroblasts	Polarization and fibrosis
IL-6	Reactive cholangiocytes	Cholangiocytes, HSCs	Proliferation and inflammation
CXCL chemokines	Reactive cholangiocytes	Immune cells	Recruitment
Integrin αvβ6	Injured cholangiocytes	Latent TGF-β1	TGF-β activation
YAP/TAZ (mechanical; context-dependent)	Cholangiocytes, fibroblasts	Cholangiocytes, fibroblasts	Mechanosensing/stiffness-responsive signaling

CCL2, C-C motif chemokine ligand 2; CCR2, C-C chemokine receptor type 2; CSF1, colony-stimulating factor 1; CSF1R, colony-stimulating factor 1 receptor; TGF-β, transforming growth factor-beta; HSCs, hepatic stellate cells; ECM, extracellular matrix; PDGF-BB, platelet-derived growth factor-BB; Shh, Sonic hedgehog; Ihh, Indian hedgehog; SPP1, secreted phosphoprotein 1; IL-6, interleukin-6; CXCL, C-X-C motif chemokine ligand; YAP/TAZ, yes-associated protein/transcriptional coactivator with PDZ-binding motif.

## 7 Disease-specific manifestations of the ductular fibrogenic niche

Primary sclerosing cholangitis (PSC) is the paradigm of a ductular fibrogenic niche. The disease features concentric peribiliary fibrosis (“onion-skinning”), a florid ductular reaction, and prominent cholangiocyte senescence, all organized around inflamed bile ducts (37, 38). Cholangiocyte-derived chemokines such as CCL24 have been directly implicated in PSC-associated biliary fibrosis, and single-cell and spatial analyses reveal a periductal niche enriched in reactive cholangiocytes, T cells, and scar-associated macrophages (39, 40). PSC thus exemplifies how an epithelial injury response is organized—literally, in concentric rings—into a niche that drives the signature fibrosis of the disease.

Primary biliary cholangitis (PBC) illustrates a complementary facet. In PBC, autoimmune-mediated cholangiocyte injury and apoptosis drive ductopenia, yet this is accompanied by a ductular reaction that correlates with fibrosis (39, 41). Cholangiocyte senescence is a defining feature, and the SASP is thought to perpetuate portal inflammation and fibrosis even as the biliary tree is progressively depleted (11, 33). PBC therefore demonstrates that cholangiocyte loss and reactive proliferation coexist, and that the surviving reactive cholangiocytes continue to generate a fibrogenic niche even while the total biliary epithelium shrinks—a dissociation between regeneration and fibrosis that underscores the maladaptive nature of the niche.

Biliary atresia extends the niche concept to the neonatal liver. This cholangiopathy features a progressive inflammatory obliteration of the extrahepatic bile ducts, accompanied by an exuberant intrahepatic ductular reaction and rapid biliary fibrosis (42, 43). The speed and intensity with which the niche is established in infants—where epithelial injury, immune activation, and mesenchymal response coincide in a still-developing liver—reinforce that the fibrogenic niche is not a late-stage phenomenon unique to adult disease but a general and rapidly inducible program.

Metabolic dysfunction-associated steatohepatitis (MASH) is particularly instructive because it demonstrates that the niche is not restricted to cholestatic disease. In MASH, ductular reaction emerges as fibrosis advances and is now recognized as a key correlate of fibrogenesis rather than an incidental finding (1, 7). Single-cell and spatial studies map TREM2-positive and CD9-positive macrophages and activated HSCs around reactive ductules in steatohepatitic fibrosis (4, 5, 21). The convergence of metabolic injury, macrophage accumulation, and ductular reaction in MASH suggests that, once a certain threshold of chronic injury is crossed, the same spatial niche assembles regardless of whether the primary insult is biliary or metabolic.

Alcohol-associated liver disease and chronic viral hepatitis complete the spectrum. Here ductular reaction appears in advanced fibrotic and cirrhotic stages, where the periductular niche merges with broad bridging fibrosis (1, 7). While less cholangiocyte-centric than PSC and PBC, these diseases share the same terminal niche architecture—reactive ductules, scar-associated macrophages, and activated myofibroblasts—supporting the generality of the framework (4). Table 3 summarizes these disease-specific manifestations, including the primary insult, defining niche features, and characteristic fibrosis pattern in each condition.

**Table 3:** Disease-specific features of the ductular fibrogenic niche.

Disease	Primary insult	Niche features	Fibrosis pattern
Primary sclerosing cholangitis	Biliary inflammation and injury	Concentric peribiliary niche; CCL24; T cells and scar-associated macrophages	Onion-skin peribiliary fibrosis
Primary biliary cholangitis	Autoimmune cholangiocyte injury	Cholangiocyte senescence and SASP; ductopenia with reactive proliferation	Portal fibrosis with ductopenia
Biliary atresia	Neonatal cholangiopathy	Exuberant, rapidly assembled niche	Rapid biliary fibrosis
MASLD/MASH	Metabolic injury	Late ductular reaction; TREM2+/CD9 + macrophages and HSCs	Periportal and bridging fibrosis
Alcohol/viral hepatitis	Toxic or viral injury	Late-assembling niche merging with bridging fibrosis	Bridging fibrosis and cirrhosis

CCL24, C-C motif chemokine ligand 24; SASP, senescence-associated secretory phenotype; MASLD, metabolic dysfunction-associated steatotic liver disease; MASH, metabolic dysfunction-associated steatohepatitis; TREM2, triggering receptor expressed on myeloid cells 2; CD9, cluster of differentiation 9; HSCs, hepatic stellate cells.

Across these diseases, the niche is therefore expressed in graded fashion: a cholangiocyte-dominant niche in PSC and PBC, an early and rapid niche in biliary atresia, and a late-assembling niche in MASH and alcoholic/viral disease. This disease-specific variation is itself informative—it suggests that the niche is a conserved downstream program whose tempo and cellular emphasis are set by the primary insult, a point we return to when considering the outstanding questions.

## 8 Spatial omics and emerging technologies redefining the ductular niche

The recent reconceptualization of ductular reaction owes much to single-cell RNA sequencing (scRNA-seq). This technology has resolved the cellular composition of the fibrotic niche and its dynamic states—reactive cholangiocytes, macrophage subsets, and mesenchymal heterogeneity—across human liver disease (4, 21, 29). It established that the niche is a composite of transcriptionally distinct cell states, not a single pathological cell type, and it supplied the first unbiased catalogs of the ligands and receptors that these states express.

Spatial transcriptomics adds the positional information that scRNA-seq discards. Technologies such as Visium and MERFISH reveal that the relevant cell states are physically co-localized in periductular and portal zones, and studies of healthy and fibrotic human liver at single-cell resolution have mapped reactive cholangiocytes and scar-associated macrophages into defined spatial neighborhoods (5, 18, 44). This is the decisive step for the niche concept: it shows not merely that a cell state exists, but that it occupies a specific position relative to others.

Multiplex imaging platforms—CODEX, imaging mass cytometry, and multiplex immunofluorescence—extend this resolution to the protein level, validating ligand–receptor co-localization and cell-neighborhood architecture *in situ* (5). These tools confirm that the molecular interactions inferred computationally occur at actual cell–cell contacts, closing the gap between predicted and observed communication.

Reductionist systems complement these observational maps. Cholangiocyte organoids recapitulate reactive cholangiocyte biology and can even repair bile ducts after transplantation, while branching organoid and liver-on-chip models reconstruct periportal architecture and biliary fibrosis *in vitro* (45, 46). These systems allow causal interrogation of niche interactions—perturbing one compartment and observing the response of the others—something observational omics cannot resolve.

Finally, integrative computational approaches—ligand–receptor analysis, cell–cell communication inference, and niche-detection algorithms—transform these datasets into testable models of interaction (5, 21). The field has thereby moved from asking “who expresses what” to asking “who communicates with whom, and where.” That positional-communication question is, in essence, the spatial-niche concept made operational.

## 9 Therapeutic opportunities: targeting the ductular fibrogenic niche

If the niche—rather than any single cell type—sustains ductular-associated fibrosis, then the therapeutic goal shifts from eliminating a cell population to disrupting the niche's organization and signaling. We frame the emerging opportunities accordingly.

Targeting reactive cholangiocytes. Modulating the reactive cholangiocyte phenotype—its proliferation, senescence, and secretome—could de-escalate the niche at its hub (6, 11). Senolytic approaches have emerged as a potential strategy for reducing senescence-associated signaling, although their efficacy and selectivity toward senescent cholangiocytes in chronic liver disease remain to be established (34). The challenge is to blunt the maladaptive phenotype without abolishing the regenerative capacity of the biliary epithelium.

Blocking macrophage recruitment or activation. CCL2–CCR2 blockade limits monocyte-derived macrophage recruitment and has been pursued in fibrosis, on the logic that interrupting the chemokine gradient severs the immune arm of the loop (12, 17). Modulating scar-associated macrophage activation offers a complementary lever, aiming to reorient rather than eliminate the niche's macrophages (4).

Targeting fibroblasts and intercellular signaling loops. Interfering with portal fibroblast and HSC activation—including their TGF-*β*- and PDGF-driven differentiation—directly targets the matrix-producing arm (24, 25). A complementary strategy is to disrupt key feed-forward interactions themselves: CCL2–CCR2, TGF-*β* (including *α*v*β*6-mediated activation), PDGF, Hedgehog, and integrin-linked mechanotransduction represent candidate nodes that may weaken niche persistence (14, 15, 31, 32). Because several of these interactions are enriched or preferentially operative within the periductular microenvironment, spatially informed targeting may ultimately improve antifibrotic selectivity while preserving broader regenerative functions.

Spatially targeted therapies. Looking forward, the goal is not simply to deplete one cell type but to reshape the pathological spatial microenvironment—to restore a periductular milieu permissive to regeneration rather than fibrosis. Spatial omics-guided targeting of specific niche neighborhoods, rather than cell types, represents the conceptual frontier of this approach (5).

## 10 Outstanding questions and future directions

Several questions must be answered before this framework can be translated. First, when does the ductular reaction tip from regenerative response to pro-fibrotic driver, and what molecular switch determines that transition? Second, which cholangiocyte subpopulations constitute the true pathogenic population of the niche, and can they be distinguished from regenerative ones? Third, what are the relative contributions of portal fibroblasts versus hepatic stellate cells across different diseases and injury types? Fourth, is the spatial niche composition disease-specific, or does it converge on a common fibrogenic architecture? Fifth, can the fibrogenic niche be precisely disrupted without impairing the regenerative capacity essential for recovery? Answering these questions will require longitudinal, spatially resolved, and causally perturbed studies—not simply richer descriptive atlases.

## 11 Discussion

We propose a three-tiered reinterpretation of ductular reaction. First, it should not be regarded merely as a histological hallmark or a passive marker of disease activity. Second, it is a dynamic spatial niche maintained by epithelial–immune–mesenchymal interaction, in which reactive cholangiocytes, macrophages, and matrix-producing cells sustain one another through chemokines, TGF-*β*-centered signaling, ECM, and senescence. Third, this niche may represent the critical inflection point at which chronic injury shifts from regeneration to fibrosis—a transition whose control is of direct therapeutic relevance.

Several limitations temper this framework. Much of the spatial evidence derives from mouse models, and temporally resolved human data remain scarce; the field still lacks longitudinal maps that follow a single niche as it assembles, matures, and either resolves or becomes fixed. Niche composition likely differs across etiologies, and extrapolating from one disease to another—or from rodent to human—should be done cautiously. Most importantly, spatial correlation does not establish causation: co-localization of cell states does not by itself demonstrate directional, functional interaction.

Establishing causation will require perturbation studies—in organoid, co-culture, and *in vivo* systems—that selectively ablate or modulate one compartment of the niche and measure the response of the others, guided by the omics maps already available. Only such experiments can distinguish the causal nodes of the loop from its correlative correlates, and thereby identify which interactions are worth targeting therapeutically.

## 12 Conclusion

Ductular reaction is far more than the reactive proliferation of bile ductules. It is a spatially organized, self-sustaining fibrogenic niche assembled from reactive cholangiocytes, macrophages, and matrix-producing mesenchymal cells, held together by feed-forward signaling loops that span chemotaxis, TGF-*β*-centered fibrogenesis, matrix mechanics, and senescence.

Single-cell and spatial technologies now make this organization measurable and, increasingly, therapeutically addressable. Targeting the pathological organization of the ductular fibrogenic niche—rather than individual cell types alone—may provide a new therapeutic framework for chronic liver fibrosis, one that seeks to restore a regenerative microenvironment rather than merely to suppress its cellular inhabitants.
